# Youth accessing reproductive health services in Malawi: drivers, barriers, and suggestions from the perspectives of youth and parents

**DOI:** 10.1186/s12978-018-0549-9

**Published:** 2018-06-19

**Authors:** Andrew Self, Samuel Chipokosa, Amos Misomali, Tricia Aung, Steven A. Harvey, Mercy Chimchere, James Chilembwe, Lois Park, Chrissie Chalimba, Edson Monjeza, Fannie Kachale, Jameson Ndawala, Melissa A. Marx

**Affiliations:** 1 Department of International Health, Johns Hopkins Bloomberg School of Public Health, 615 N. Wolfe Street, Baltimore, MD 21205 USA; 2 National Statistical Office, Zomba, Malawi; 3 Lilongwe District Health Office, Ministry of Health, Lilongwe, Malawi; 4 Reproductive Health Directorate, Ministry of Health, Lilongwe, Malawi; 5 Department of Nutrition, HIV and AIDS, Ministry of Health, Lilongwe, Malawi; 6 Malawi College of Health Sciences, Blantyre, Malawi

**Keywords:** Adolescent, Youth, Malawi, Qualitative research, Sexual and reproductive health, Contraception, Family planning

## Abstract

**Background:**

Malawi has made progress in increasing its overall modern contraceptive prevalence rate since 2000, resulting in a dramatic reduction in its total fertility rate. However, youth, 15–24 years, have not had the same successes. Teenage pregnancies are on the rise and little progress has been made in reducing unmet need for family planning among youth. With two-thirds of the population under the age of 25 and with Malawi’s rapid population growth, reducing unmet need for family planning among youth remains a priority for the government’s reproductive health agenda. To further explore this situation, we conducted a qualitative study to explore the perspectives of youth and adults about the drivers and barriers to youth accessing family planning in Malawi and their ideas to improve services.

**Methods:**

We conducted 34 focus group discussions with youth aged 15–24 and parents or legal guardians of female youth in 3 districts in Malawi. Focus groups were translated and transcribed. Data was input into Dedoose and analyzed using a thematic framework to identify broader patterns and themes.

**Results:**

Youth participants felt motivated to use family planning to protect themselves from sexually transmitted diseases and to prevent unwanted pregnancies. Females focused on the consequences of unplanned pregnancies and believed family planning services were targeted primarily at them, while males thought family planning services targeted males and females equally. Barriers to youth accessing family planning included contraception misconceptions, the costs of family planning services, and negative attitudes. Parents had mixed views on family planning. While many parents acknowledged they could play a role in supporting youth, most said they are reluctant to support youth using family planning. Participants said improving counseling services, integrating family planning services and education within school curricula, and utilizing youth clubs could improve family planning services for youth.

**Conclusions:**

Policy makers and program implementers should consider the diverse preferences among youth and parents and continue seeking their input when designing policies and programs. Youth clubs and school-based services were among the most common suggestions. However, the effectiveness of youth clubs and school-based initiatives to increase contraceptive use among youth in Malawi is not clear.

**Electronic supplementary material:**

The online version of this article (10.1186/s12978-018-0549-9) contains supplementary material, which is available to authorized users.

## Plain English summary

Malawi, a landlocked country of 18 million people in Southeastern Africa, has made progress in several development goals over the past decade. Sexual and reproductive health is one area Malawi has seen progress in recently. For example, the average number of children a woman is expected to have over her lifespan decreased from 5.7 children in 2010 to 4.4 children in 2015. Overall, women saw these improvements, however youth 15–24 years old, experienced slower progress and teenage pregnancies increased over that time period. To investigate these differences in sexual and reproductive health outcomes we conducted focus group discussions with youth and adults about the drivers and barriers of youth accessing family planning in Malawi and their ideas to improve services. Data were collected in July and August 2016 in Dowa, Machinga, and Phalombe districts. Youth felt motivated to use family planning to protect themselves from sexually transmitted diseases and to prevent unwanted pregnancies. Females focused on the consequences of unplanned pregnancies and believed family planning services were targeted primarily at them, while males thought family planning services targeted males and females equally. Barriers to youth accessing family planning included contraception misconceptions, costs of family planning services, and negative attitudes. Participants said involving community leaders in family planning discussions, improving counseling services, integrating family planning services and education within school curricula, and utilizing youth clubs could improve family planning services. These findings can be used to inform family planning programming for youth and to craft more youth inclusive and responsive policy.

## Background

Malawi reduced its total fertility rate (TFR) dramatically from 5.7 in 2010 to 4.4 in 2015 [1, 2]. While reductions in fertility were seen in all age groups over this period, reductions in age-specific fertility rates (ASFR) among women aged 15–19 only decreased by 11% (152 to 136 per 1000 births) compared to at least a 19% reduction for all other 5-year age groups of women, during the same time period. Women ages 20–24 have the highest ASFR among all age groups in Malawi. Additionally, the percentage of women ages 15–19 who have begun childbearing rose from 25.6 to 29.0 in the same period, and this age group has the highest unmet need for contraception among sexually active women of reproductive age [1].

As of 2015, Malawi reduced its child mortality by two-thirds compared to its 1990 level, mostly due to better management of childhood diseases, improved vaccination coverage, and more effective prevention and treatment of HIV and malaria [3]. These improvements in child survival, combined with a lag in reductions in TFR in this early stage of the demographic transition led to an ‘adolescent bulge’ in Malawi [4]. As of 2015, two-thirds of Malawi’s population was under the age of 25 [1]. As this group ages, government officials are concerned about the country’s ability to meet the environmental, educational, and health care needs of the projected population [4, 5] and have been paying increasing attention to meeting reproductive health needs of youth. In fact, in addition to reducing unwanted pregnancies increasing the modern contraceptive prevalence rate (mCPR) among youth (youth refers to those 15–24 years old as defined by the United Nations) can improve child spacing, decrease adverse birth outcomes, reduce unsafe abortions, and improve schooling for girls [6–9].

### Youth friendly family planning services

Youth friendly health services (YFHS) are meant to provide youth with equitable, effective, accessible, acceptable, and appropriate health services [10], since youth have developmental needs that may not be met by standard health services [11, 12]. Offering youth-friendly family planning (FP) services as a key element of YFHS can increase mCPR among youth [11, 13, 14]. Malawi started providing FP to youth in 2000 and created its first YFHS program in 2007 [15]. A recent study found that 68% of health center providers had been trained in YFHS and only 63% of those trained in YFHS were trained in contraceptive counseling. In that study youth reported facing barriers related to long waiting times, negative health provider attitudes, and a lack of confidentiality [15]. These findings led to Malawi’s 2015–2020 YFHS strategy [16]. Preliminary findings from a 2017 study on the implementation of YFHS in Malawi support findings from a 2014 evaluation that found YFHS implementation in Malawi varied by district, was implemented sporadically and relied heavily on donor support [15].

Thus, although a YFHS policy exists in Malawi, the availability and acceptability of the services provided are largely unknown. This qualitative study was conducted as part of a multifaceted, phased mixed-methods evaluation on youth-friendly FP services to explore barriers and facilitators to access and utilization of FP services in Malawi.

## Methods

### Research design

This qualitative study used semi-structured focus group discussions (FGD) to elicit perspectives and norms about youth-friendly FP services in Malawi to allow consistency in the topics discussed but also make room for additional thoughts and topics to emerge during the discussion.

### Selection of study sites and participants

The study took place in three of the 28 districts of Malawi, Dowa, Machinga, and Phalombe in July 2016. We purposively selected these districts based on: (1) variation in TFR and ASFR from the 2010 Demographic Health Survey (the 2015–2016 Malawi Demographic Health Survey was not yet available) and FP service quality from the 2013 Service Provision Assessment; (2) variation in the non-governmental organizations (NGOs) providing FP services; and (3) for geographic accessibility. We selected two facility catchment areas from each district for study recruitment and worked with the district FP Coordinator, Health Surveillance Assistants (HSA) and NGO staff to recruit participants, since they were well connected to the community and could recruit participants based on the study’s screening criteria. We purposively selected FGD participants and included parents/guardians of female youth, and youth by age, sex, and by school and marital status.

### Data collection methods

A team of 5 adult data collectors from the National Statistical Office (NSO); the Department of Nutrition, HIV and AIDS; the Reproductive Health Directorate and two district Health Offices conducted the FGDs. They were trained in the research protocol, ethics, qualitative interviewing techniques, and the consent process. They conducted all FGDs in Chichewa using translated guides (guides are available as Additional files 1 and 2). Focus group discussions had between 5 and 10 participants and took between 50 and 110 min. One moderator and one note-taker, who captured non-verbal communication and participant demographics, conducted each FGD in a private location organized by the HSA or NGO assisting with recruitment. For in-school youth FGDs some HSAs/NGOs worked with local teachers to recruit youth. We asked the HSA or NGO assisting with recruitment to pre-screen all participants. During the assent/consent process the data collection team screened all FGD participants to ensure they met the study’s inclusion criteria. Youth FGDs were organized by the age, sex, and marriage and school status of the participants. For youth FGDs, the moderator was of the same sex as the youth to help create a more open environment [17]. Female youth FGDs were divided by age, school, and marriage status based on recommendations from consultation with the Malawian members of the study team. Male youth FGDs were divided by age and school status, not marriage status, also based on recommendations from consultation with the Malawian members of the study team. For logistical reasons we conducted the parent/legal guardian FGDs with parents of female youth, since the parents had to come to provide consent for their child. We conducted those FGDs in a different location, but at the same time. We audio-recorded all FGDs. Daily debriefings were held after data collection activities to discuss emerging themes and topics, and areas to improve or follow-up on in subsequent FGDs.

### Ethics

The Johns Hopkins Bloomberg School of Public Health Institutional Review Board reviewed and approved the study. The Malawi National Health Science Research Committee waived the study from full review, considering it exempt. We informed participants about the study, and asked for consent to be indicated by initials or a thumbprint. Parents/guardians of minors (aged 15–17 who were not married or emancipated) provided informed consent, and the minors participated only after assenting.

### Data management and analysis

All FGDs were translated and transcribed verbatim into English. A selection of the transcripts was checked further for transcription and translation accuracy. We developed a codebook using a team-based method [18] with a combination of codes defined *a priori* from our research questions along with open or initial coding, an approach borrowed from grounded theory where codes emerge from the data [19]. Dedoose [20] was used for coding and data management. It allowed for collaboration among the study team members in Malawi and Baltimore. We used inter-rater agreement indicators to identify and resolve differences in the coding process. We identified broader themes and patterns within and among the different participant groups, geographic locations, and relevant demographic characteristics (e.g., school status) using the framework analysis method [21].

Youth and parents’ suggestions for how to improve FP services for youth were arranged into 5 thematic areas: institution, health provider conduct, service delivery, FP education and information, and parents and society. Suggestions within each theme were organized from the most to least common among male youth, female youth, and parents.

## Results

We held 34 FGDs with 255 youth and 40 parent/guardian participants (Table 1). We sought both female and male parents/guardians for the parent FGDs, but no male parents or guardians participated. While we selected parents of female youth for logistical reasons, we did not exclude parents of male youth, and parent participants had an average number of 4.3 children. Among out-of-school males both married and single males participated.

**Table 1:** Number of FGDs and participants by participant type

Participant type	Number of FGD	Number of FGD participants	Total participants
Female Youth			
In-school (15–17)	6	10, 10, 8, 10, 9, 8	55
Out-of-school & unmarried (15–24)	5	7, 9, 10, 10, 6	42
Married (15–24)	6	10, 10, 10, 10, 10, 9	59
Male Youth			
In-School (15–17)	5	10, 10, 10, 9, 7	46
Out-of-School (15–24)	6	10, 8, 10, 9, 10, 6	53
Parents/legal guardians	6	6, 6, 6, 5, 9, 8	40
**Total**	**34**		**295**

### Drivers of youth accessing family planning services

Societal benefits and personal protection emerged as the main motivators for youth to use FP. The perceived societal benefits of using FP included: managing population growth; reducing demand for public services; and reducing population-related adverse effects, such as food and water shortages, environmental degradation, and unsustainable pressure on the government to provide public goods and services.

*“When the population grows, we are faced with a lot of challenges… food becomes scarce. Some kids are stunted because of this.”*(Female, out-of-school, 15–18 yrs., Machinga).

Participants expressed the need for youth to protect themselves and avoid negative consequences from unprotected sex as another motivating factor. Participants mentioned preventing unwanted pregnancies, avoiding birth complications as a result of adolescent pregnancies and improper child spacing; fistulas, not wanting to die at an early age as a result of HIV, and protection from sexually transmitted diseases as drivers for youth accessing FP.

*“When you want to sleep with a woman you must use a condom to prevent contracting the virus.”*(Male, out-of-school, 15–20 yrs., Phalombe).

Consequences expressed by participants, especially female youth, extended beyond health concerns to include the repercussions of unplanned pregnancies such as loss of educational opportunities, early marriage, and social scorn. While both males and females expressed these concerns, female youth felt most affected, and believed they had to bear the responsibility of protecting themselves.

*“If we use contraceptives we can have a manageable number of children, rather than having so many that we can’t raise them. Through contraceptives the woman has time to raise her child, and the child can grow healthily.”*(Female, married, 18–24 yrs., Dowa).

*“Girls are the ones who carry the burden of child birth. The man can walk around freely and claim that he has no children, while girls cannot, they have to carry the baby on their back.”*(Female, out-of-school, 15–18 yrs., Machinga).

Most male and parent participants thought females were more encouraged to use FP. Only a few male youth and parents, and no female youth thought males and females were encouraged equally. In response to questions about whether males or females are more encouraged or if there is a difference in encouragement to use family planning a variety of responses were given:

“*No its all the same, family planning is very important to everyone.”*(Male, out-of-school, 15–20 yrs., Dowa).

Female youth*: “Yes [there is a difference in encouragement], they want the girls should finish school.”*

Moderator: *“What about the boys?”*

Female youth: *“They don’t encourage them, they know that once a boy impregnates a girl, he can still continue with school.”*(Female, in-school, 15–17 yrs., Machinga).

*“There is no difference [in encouragement to use FP], both boys and girls use contraceptives.”*(Parent, 20–52 yrs., Machinga).

### Barriers youth face accessing family planning services

#### Misconceptions and perceived side-effects

Most participants could name popular contraceptive methods and could list which methods were available in their community, although misconceptions about how contraceptives work and their side effects were common (Table 2). The most frequently mentioned misconceptions were that the use of contraceptives cause permanent sterility, illness, cancer, and weaken men’s libido. Participants had the fewest misconceptions about condoms and the most misconceptions about oral contraceptive pills. Male youth and out-of-school youth were more likely than female and in-school youth, and parent participants to talk about contraceptive misconceptions. Youth expressed a preference for condoms over other contraceptive methods because of the perceived side effects.

*“For us youth there are [contraceptives] which we can take, and there are others which we cannot take as they can bring problems on our lives. The youth mainly use condoms, that one cannot bring problems unlike methods like IUD. People even fall sick because of such methods.”*(Female, in-school, 15–17 yrs., Machinga).

**Table 2:** Perceived risks and side-effects of contraceptives reported by youth and parents

Method	Male youth	Female youth	Parents/guardians
Male condoms	• If expired can cause infections	• Oils/lubricants cause stomach pains	• Cause cancer and sores on penis
	• Lubricants cause cancer and sores on penis		
	• Worms in the packaging		
	• Cause illness		
Oral contraceptive pills	• Cause permanent sterility	• Pills clog up and accumulate in abdomen	• Weaken sperm cells
	• Weaken the man’s libido	• Cause illness	• Pills clog up and accumulate in abdomen
		• Ruin the inside of a person, harms the uterus	
	• Cause illness and death		
	• Stops egg production permanently		
	• Make the woman unattractive		
	• Encourage promiscuity		
	• Men think women are less satisfying sexually		
	• Cause continuous menstruation in women		
Depo-Provera (injectable)	• Cause permanent sterility	• Cause permanent sterility	• Cause permanent sterility
	• Damage the ovaries and destroys egg cells	• Pain in the heart, arms, and legs	• Weaken the man’s libido
	• Weaken the man’s libido	• Cause illness	• Stomach pains
	• Prevent women from getting cancer	• Weaken the man’s libido	• Skin glows and women look healthy
	• Cause illness		
	• Cause sperm accumulation in women’s body		
	• Women are not as ‘sweet’ sexually		
	• Women get enlarged breasts		
Implants	• Cause permanent sterility	• Cause permanent sterility	
	• Move around body and cause illness	• Cause birth of twins after discontinuation	
	• Damage nerves and blood vessels	• Damage reproductive organs	
	• Cause uterine cancer	• Heart and body pains	
	• Continuous menstruation in women	• Close the birth canal	
IUDs	• Cause permanent sterility	• Cause illness	
	• Cause birth complications		
	• Can cause death

#### Costs

Participants also said costs were a barrier to use of contraceptives. Some NGO providers regularly charge for certain services. Most youth said that they prefer government providers over NGO providers largely because they are supposed to be free. However, participants in one district reported that some government providers charged fees. Additionally, both male and female youth mentioned transport costs and long distances as another barrier to seeking FP services. Many youths wanted FP services closer in their communities because of the distances and transport costs, while other youth were okay with services being further away, since the distance protected their privacy.

*Female 1: “These [government FP] services are there to assist the youth and they are asking us to pay, but most of us do not have money.”*

*Female 2: “So one may want to have these services but because of the fee that is attached to it they fail, and in the process become pregnant and drop off from school.”*(Females, out-of-school, 15–18 yrs., Phalombe).

*“It is discouraging to go to the hospital and find a lot of people waiting and also considering the money you have to use for transport….”*(Male, out-of-school, 19–24 yrs., Machinga).

#### Societal attitudes towards family planning

Lastly, participants said negative attitudes about youth using FP are a major barrier. All participants thought parents expressed negative opinions of youth using FP and parents could prevent youth from accessing FP services. However, the majority of youth also said parents provided FP support and information especially around abstinence. A few parent participants acknowledged that they could play a role in encouraging youth to use FP, but also noted that many parents are reluctant to support youth using FP.

*“Parents should give guidance to the child to stop taking family planning methods and advise her to stop being involved in sexual intercourse… if they don’t listen we tell them to go for family planning methods.”*(Parent, 35–53 yrs., Dowa).

*Parent 1: “[Parents] tell [their children] that it’s not good to bear children frequently so they should use the family planning methods.”*

*Parent 2: “Other youth believe that you cannot eat a sweet while it is in its wrapper, hence they can’t use a condom, so we advise them that that belief is not good.”*(Parent, 23–58 yrs., Phalombe).

According to youth participants a minority of health providers decline to assist youth with proper FP services in part because they are too young and/or unmarried.

*“If you go seeking for contraceptives but look very young [health providers] act as if they do not want to help us. They do not serve boys and girls who have not reached 18 years of age.”*(Male, in-school, 15–17 yrs., Dowa).

Some parents also said youth below age 18 are not old enough to be sexually active and therefore do not need FP and that youth should focus on completing their education and not engage in sexual activities.

*“When you tell [youth] to use contraceptives, you give them the opportunity to indulge in sexual intercourse and they lose sight of school since they are using contraceptives.”*(Parent, 20–52 yrs., Machinga).

Peers were mentioned as resources to support other youth if they shared news and information about FP, but they were also reported to sometimes mock and tease peers who they knew wanted to use FP.

Youth said they want confidential and safe spaces, and that privacy and confidentiality are often lacking where services are offered. For example, participants said they have to queue together with older clients. Both male and female youth reported that health providers or other clients were known to report youth to their parents, resulting in fear of going to facilities to access FP services. To avoid these breaches of confidentiality, some youth said they prefer to access services from health facilities, where other patients would not know what services they were seeking, over community health workers from their communities or outreach activities that are specifically for FP. Additionally, some female youth preferred longer-acting methods to avoid frequenting health facilities.

*“We do this [get an implant] because we don’t want to go to the hospital. When we go to the hospital the whole world knows… we are using these contraceptives. So when girls show up to the hospital, they feel ashamed.”*(Female, out-of-school, 19–23 yrs., Machinga).

*“When they see someone going for contraceptives, it becomes a big issue which is why youths are scared to use contraceptives. When someone has been seen going to such facilities, it becomes an issue at home… so the youths are very scared and if it is known that youths are on contraceptives, they would be resented by their parents.”*(Parent, 20–52 yrs., Machinga).

### Suggestions from participants for improving family planning services

The most common suggestion (Table 3) among youth participants was the formulation of youth clubs, where youth could share FP information and health providers could offer FP counseling, education, and commodities. The second most common suggestion among youth participants and the most common among parents was the need for more FP counseling for youth. Participants said this counseling would ensure youth understand the importance of FP and how methods work.

**Table 3:** Participants’ suggestions by theme

Suggestion theme	Male youth	Female youth	Parents
Institutional	1. More providers for FP services	1. More youth specific days/times for FP provision	1. Youth specific rooms for FP provision
	2. More youth specific days/times for FP provision		
	3. Use feedback from clients		
Health provider conduct	1. Give more detailed FP counseling	1. Give more detailed FP counseling	1. Give more detailed FP counseling
	2. Ensure confidentiality	2. Ensure confidentiality	2. Ensure confidentiality
	3. Avoid judgmental attitude towards youth	3. Do not demand fees for FP services	
Service delivery	1. Ensure reliable supply of FP commodities	1. Utilize youth clubs	1. Community-based delivery
	2. Utilize youth clubs	2. Community-based delivery	2. Utilize youth clubs
	3. Community-based delivery	3. More government/NGO partnerships	3. Ensure reliable supply of FP commodities
	4. FP integrated into recreational activities	4. Ensure reliable supply of FP commodities	4. FP integrated into recreational activities
	5. More government/NGO partnerships	5. FP integrated into recreational activities	
FP education and information	1. FP information and provision in schools	1. Conduct more community sensitization	1. FP education via peer networks
	2. FP education via peer networks	2. Health education with parents and leaders	
	3. Conduct more community sensitization	3. FP information and provision in schools	
	4. Health education with parents and leaders	4. FP education via peer networks	
Parents and society	1. Parents should be more supportive	1. Providers, parents, and community leaders should dispel FP misconceptions	1. Parents and the community should be more open towards youth about FP
	2. Involve community leaders in FP talks	2. Parents and the community should be more open towards youth about FP	
		3. Involve community leaders in FP talks	

## Discussion

Youth and parents reported that health risks and side effects of contraception, negative attitudes towards FP, a lack of privacy, fear of being exposed for using FP, and costs were key barriers preventing youth from accessing FP. Protection from infections and unplanned pregnancies drive youth to use FP services.

Youth and parents suggested adding youth-specific spaces and times for FP provision, youth clubs, better counseling services, and FP provision and information in schools. Despite these suggestions, the evidence of some interventions is mixed. In a previous study, youth clubs in Machinga district were associated with improved FP knowledge but they did not find differences in contraceptive use among club participants and non-participants [22]. Furthermore, according to a review of 18 youth center programs, the programs benefitted a minority of the target population whom tended to be male, older, and more educated [23]. Incorporating FP information in schools, especially comprehensive sexuality education, can be effective when it includes a focus on gender and power relations [24, 25]. Malawi’s 2015–2020 YFHS strategy aimed to establish and strengthen ‘safe spaces for youth’ (i.e. youth clubs) and increase access to comprehensive sexuality education. But societal, political, and funding pressures can affect the content and quality of school and youth-focused programs.

As with previous studies [1, 26, 27], awareness of the types of contraceptives appeared high overall in our study, but accurate understanding of the mechanism and side effects associated with specific methods were low, and misconceptions were common [28, 29]. Better FP information is needed for both youth and their parents to address the misconceptions identified. In fact, better counseling was among the most common suggestion to improve FP services for youth.

Research has shown that there are additional challenges reaching out-of-school females with FP services [24, 30]. Our study found that out-of-school youth held more misconceptions about FP than in-school youth. This signals that there are differences in FP knowledge among in-school and out-of-school youth. Therefore, targeted strategies are needed to reach out-of-school female youth to improve their knowledge about contraceptives and meet their demand.

Our findings are consistent with other qualitative [31] and quantitative [26] studies pointing to the lack of privacy and confidentiality as major barriers to use of FP among youth. Youth are afraid of being reported to their parents. The need to avoid repeated trips to the clinic for FP drove youth to get implants and other long acting contraceptives, highlighting the importance for health providers to have a variety of FP methods available to youth. More private spaces are needed for youth access services.

We also found that FP messages target females more than males. While this focus may motivate some female youth to seek services, because men often control the finances they also make key family planning and sexually-transmitted disease prevention decisions [32, 33]. Even among youth, unmet need can be reduced when men are encouraged to discuss FP with their partners and facilitate FP care-seeking [34]. A 2011 study on the *Male Motivator Project* found that male targeted messaging using peer networks increased contraceptive use in the study area in Malawi [35].

In addition to including males in FP discussions, including parents and guardians in communication about FP topics with their children and the community could help improve FP outcomes for youth. As we found, parents can be supportive of FP, but they often would not promote their children to use FP. Parent-child dialogue has been shown to be associated with some behavioral outcomes for youth, such as reduced sexual activity and FP use among youth [36]. Working with parents and pushing for more open dialogue around youth FP use could help reduce some of the socio-cultural barriers youth face.

Youth in one district reported that they had to pay to receive FP from government health providers, even though FP services are supposed to be free in Malawi. These costs are especially problematic for female youth since they are less likely to be employed than male youth and have little access to financial resources. Female youth in rural, resource-limited settings are most at risk of unwanted pregnancies, have higher fertility rates, and marry younger and are therefore most in need of public FP services. We selected this district because it had high TFR, high ASFR among 15–19 and 20–24 year olds [2], and low quality of FP counseling services according to Quick Investigation of Quality indicators [37] derived from the 2013 Service Provision Assessment [38]. While our findings cannot be linked directly to high TFR, high ASFR or low FP quality, they raise questions about whether informal fees could be related to the worse outcomes. To address the barrier of cost on the supply-side the Ministry of Health needs to ensure that all public providers are following government protocol providing free FP services and continue working with NGOs to provide affordable or no-cost FP services, especially for at risk youth.

Our study captured the perspectives of parents and youth disaggregated by age, sex, and marriage status. This allowed us to make within and across group comparisons based on those demographics. But our study also has several limitations. We explored the topic in 3 out of 28 districts in Malawi. We did not select any districts in the North and we cannot compare responses by region even though regional differences often reflect cultural or religious differences [39]. Also, given the sensitive nature of FP especially among youth, participants may have been reluctant to share openly among their peers and with the study team, who were older than the youth participants. Social desirability bias may have also affected how participants discussed norms around FP. To reduce social desirability and the chances of inadvertent disclosure of sensitive information, we asked participants to provide their guesses about their peers’ perspectives rather than their own. Lastly, we sought both female and male parents/guardians of female youth for the parent FGDs, but no men participated, so we were unable to explore sex differences in parental responses.

## Conclusions

These findings can be used to inform YFHS program re-orientation, to craft more youth- inclusive and responsive policy, and to inform the design of community based interventions that work with parents and guardians, health providers, and community leaders. It is important to engage these gate-keepers, because they play a critical role in youth’s access to FP services. Furthermore, these findings point to the need to conduct more comprehensive analysis into other barriers of youth accessing FP, such as gender dynamics and training and biases affecting provision of services to youth. With a more comprehensive understanding of the reasons youth are not accessing contraception in Malawi, program implementers and policy makers can craft more effective strategies to address the family planning needs of youth.

## Additional files

Additional file 1: FGD parent guide (DOCX 18 kb).

Additional file 2: FGD youth guide (DOCX 18 kb).

## Abbreviations

ASFR: Age-Specific Fertility Rate
FGD: Focus Group Discussion
FP: Family Planning
HSA: Health Surveillance Assistant
mCPR: Modern Contraceptive Prevalence Rate
NGO: Non-Governmental Organization
NSO: National Statistical Office
TFR: Total Fertility Rate
YFHS: Youth-Friendly Health Services
