# Supplementary material for: Youth accessing reproductive health services in Malawi: drivers, barriers, and suggestions from the perspectives of youth and parents
Source: Reprod Health. 2018 Jun 19;15:108. doi: 10.1186/s12978-018-0549-9 (PMC6008927; doi:10.1186/s12978-018-0549-9)
Supplement: Supplementary file 1 — FGD parent guide (DOCX 18 kb). [file 12978_2018_549_MOESM1_ESM.docx]

**FGD – Guide for parents**

Introduction:

Thank you for taking your time to talk with us today. I’m (name) and I will be leading the focus group today. We are here to discuss youth friendly reproductive health services in Malawi. We want this to be an open discussion.

First we need to set some rules. What do you all think some rules for our discussion should be?

(Suggest these if they don’t mention them)

1. Only one person speaking at a time.
2. Respect what others have to say.
3. Don’t interrupt people.
4. Please silence all phones.
5. Keep what is said in this discussion in this discussion. Please don’t discuss what people said outside of this discussion.
6. Don’t say anything you don’t want everyone to hear.
7. Please do not use people’s names during the discussion. We don’t want any true names to be recorded.

Now that we have some rules I want to tell you how this will work. I will be moderating the discussion, so I will ask some questions and I want you all to discuss the questions and your answers. We want to hear your thoughts and opinions about the topics. This is meant to be a discussion not an interview. We want to hear from everyone so please do not be afraid to share. This audio recorder is going to record the discussion so we can transcribe and translate the discussion. My partner (name of notetaker) will be taking notes during the interview. As the moderator I will not play an active role in the discussion of topics. I will pose questions and guide the discussion, but I will refrain from joining the discussion.

Do you all have any final questions before we get started?

(Start recording)

1. Let’s start by talking about youth friendly health services. What do you all think youth friendly health services are?
   1. Have you heard of youth friendly reproductive health services before?
   2. What would make a health service youth friendly?
      1. What factors do you think would be most important to youth?
         1. These could include things like: Quality – commodity availability, friendliness, waiting times, counseling quality, privacy. Say this if people are not speaking and get their ideas about those and if they are important or not.
   3. Have you all had any experiences with these kinds of services or heard of them before?
      1. If so what were they?
      2. What do you think about them?
      3. What did you like/dislike about them?
      4. Do you think they are effective?
2. Now I want to talk a little more about youth friendly reproductive health services. Do you know what different reproductive health services are available in your community?
   1. Do you know who the providers of these services are?
      1. Do you think youth would prefer seeing one provider over another?
         1. Why would someone prefer one provider over another?
      2. How people know where to go for services?
      3. What do you think providers tell youth about these services?
         1. Do you think health providers encourage youth to use them?
   2. Do you think youth should learn about reproductive health in school?
      1. What do you think they should learn?
         1. Who should teach students about these things?
      2. Besides school who else should youth learn about reproductive health from?
         1. Friends? Parents? Youth groups? Church?
3. Next I want to talk some about contraception. I don’t want you to talk about your experiences or your children's experiences, but I want to know what you know about different contraception methods and your opinions about youth accessing contraception?
   1. What are some contraceptive methods you all know of?
      1. Probe on what they are and how they work
      2. Are these accessible to youth in your community?
         1. Probe on where available - health clinics? Schools? Friends? Pharmacies? Other stores?
         2. What places are most likely to be accessed for contraception services for youth?
      3. Do you think youth would seek these services out?
         1. Why? If no, probe on why youth would not seek these services out. If yes why would youth seek these services out?
         2. What would make youth more likely/less likely to access and utilize these services?
         3. What are the reasons why someone would not use family planning?
            1. Probe on: Method? Misconceptions (side effects, how to get pregnant, HIV)? Religious issues?
4. What role does society (parents, community, schools, church, friends) play in people’s decisions to use and access reproductive health services?
   1. What groups are supportive?
      1. Probe on how they are supportive, what makes them supportive?
   2. What groups are not supportive?
      1. Probe on how they are not supportive, what makes them not supportive?
   3. Do you all think there are differences in the level of support for:
      1. Male vs female
      2. Married vs unmarried
      3. Others?
5. What do you think a parent’s role is in teaching children about reproductive health?
   1. Should parents play an active role in teaching their children about reproductive health and family planning?
   2. Should youth be educated about family planning and reproductive health services including contraception? Whose job is it to provide this education or decide?
      1. Probe on answers, why or why not?
6. Do you all have any recommendations for how to improve reproductive health services for youth? Are there things that you believe providers are doing well for youth and should keep doing?
7. We’ve talked a lot about your roles as parents and what your thoughts about YFHS are, but lastly I want to discuss some of your beliefs and perceptions about fertility and marriage are in Malawi
   1. What do you all think should be the ideal family size in Malawi?
      1. What are the reasons for that?
   2. At what age do you all think should women start having children?
      1. What are the reasons for that?
   3. At what age do you all think should women and men start getting married?
      1. What are the reasons for that?
8. Additional Topics to probe on if they come up: questions on facility versus community based FP provision? Any supply issues (many youths get their condoms from markets, not health facilities or workers), quality of provision questions? Knowledge of, comfort using YFHS?
9. Are there any other topics you would like to discuss? Have you thought of anything else based on our discussion?

Thank you very much for taking your time to participate in this discussion today? We really appreciate your openness and insight. We will now provide you some snacks.

Demographic profile sheet

| ID | Age | Sex | Marital status | # of children | School status | Notable characteristics |
| --- | --- | --- | --- | --- | --- | --- |
| 1. |  |  |  |  |  |  |
| 2. |  |  |  |  |  |  |
| 3. |  |  |  |  |  |  |
| 4. |  |  |  |  |  |  |
| 5. |  |  |  |  |  |  |
| 6. |  |  |  |  |  |  |
| 7. |  |  |  |  |  |  |
| 8. |  |  |  |  |  |  |
| 9. |  |  |  |  |  |  |
| 10. |  |  |  |  |  |  |
