# Supplementary material for: Youth accessing reproductive health services in Malawi: drivers, barriers, and suggestions from the perspectives of youth and parents
Source: Reprod Health. 2018 Jun 19;15:108. doi: 10.1186/s12978-018-0549-9 (PMC6008927; doi:10.1186/s12978-018-0549-9)
Supplement: Supplementary file 2 — FGD youth guide (DOCX 18 kb). [file 12978_2018_549_MOESM2_ESM.docx]

**FGD – Guide for youth**

Introduction:

Thank you for taking your time to talk with us today. I’m (name) and I will be leading the focus group today. We are here to discuss youth friendly reproductive health services in Malawi. We want this to be an open discussion.

First we need to set some rules. What do you all think some rules for our discussion should be?

(Suggest these if they don’t mention them)

1. Only one person speaking at a time.
2. Respect what others have to say.
3. Don’t interrupt people.
4. Please silence all phones.
5. Keep what is said in this discussion in this discussion. Please don’t discuss what people said outside of this discussion.
6. Don’t say anything you don’t want everyone to hear.
7. Please do not use people’s names during the discussion. We don’t want any true names to be recorded.

Now that we have some rules I want to tell you how this will work. I will be moderating the discussion, so I will ask some questions and I want you all to discuss the questions and your answers. We want to hear your thoughts and opinions about the topics. This is meant to be a discussion not an interview. We want to hear from everyone so please do not be afraid to share. This audio recorder is going to record the discussion so we can transcribe and translate the discussion. My partner (name of notetaker) will be taking notes during the interview. As the moderator I will not play an active role in the discussion of topics. I will pose questions and guide the discussion, but I will refrain from joining the discussion.

Do you all have any final questions before we get started?

(Start recording)

1. Let’s start by talking about youth friendly health services. What do you all think youth friendly health services are?
   1. Have you heard of youth friendly reproductive health services before?
   2. What would make a health service youth friendly?
      1. What factors are the most important to you?
         1. These could include things like: Quality – commodity availability, friendliness, waiting times, counseling quality, privacy. Say this if people are not speaking and get their ideas about those and if they are important or not.
   3. Have you all had any experiences with these kinds of services?
      1. If so what were they like?
      2. How did they make you feel?
      3. What did you like/dislike about them?
2. Now I want to talk a little more about youth friendly reproductive health services (such as family planning). What different reproductive health services are available in your community?
   1. Do you know who the providers of these services are?
      1. Do you think youth prefer seeing one type of provider over another?
         1. Why would someone prefer one provider over another? (probe: community, facility based)
         2. Would you all prefer to go somewhere to access services or have someone bring services to you? To your school? To another location?
      2. How do youth know where to go for services?
      3. What do you think providers tell youth about these services?
         1. Do you think health providers encourage youth to use them?
   2. Do you think people learn about reproductive health in school?
      1. What do you think they learned?
         1. Who would teach students about these things?
      2. Besides school where else do youth learn about reproductive health?
         1. Friends? Parents? Youth groups? Church?
3. Next I want to talk some about contraception, I don’t want you to talk about your experiences, but I want to know what you know about different contraception methods?
   1. What are some contraceptive methods you all know of?
      1. Probe on what they are and how they work
      2. Where can someone your age access these?
         1. Probe - health clinics? Schools? Friends? Pharmacies? Other stores?
         2. What places are mostly likely to be accessed for contraception services?
      3. Do you think people your age would seek these services out?
         1. Why? If no, probe on why youth would not seek these services out. If yes why would youth seek these services out?
         2. What would make youth more likely/less likely to access and utilize these services?
         3. What are the reasons why someone would not use these services?
            1. Probe on: Method? Misconceptions (side effects, how to get pregnant, HIV)? Religious issues?
4. What role does society (parents, community, schools, church, friends) play in people’s decisions to use and access reproductive health services?
   1. What groups are supportive?
      1. Probe on how they are supportive, what makes them supportive?
   2. What groups are not supportive?
      1. Probe on how they are not supportive, what makes them not supportive?
   3. Do you all think there are differences in the level of support for:
      1. Male vs female
      2. Married vs unmarried
      3. Others?
5. Do you all have any recommendations for how to improve reproductive health services for youth? Are there things that you believe providers are doing well for youth and should keep doing?
6. We’ve talked a lot about your perceptions of YFHS, but lastly I want to discuss some of your beliefs and perceptions about fertility and marriage are in Malawi
   1. What do you all think should be the ideal family size in Malawi?
      1. What are the reasons for that?
   2. At what age do you all think should women start having children?
      1. What are the reasons for that?
   3. At what age do you al think should women and men start getting married?
      1. What are the reasons for that?
7. Additional Topics to probe on if they come up: questions on facility versus community based FP provision? Any supply issues (many youths get their condoms from markets, not health facilities or workers), quality of provision questions? Knowledge of, comfort using YFHS?
8. Are there any other topics you would like to discuss? Have you thought of anything else based on our discussion?

Thank you very much for taking your time to participate in this discussion today? We really appreciate your openness and insight. We will now provide you some snacks.

Demographic profile sheet

| ID | Age | Sex | Marital status | # of children | School status | Notable characteristics |
| --- | --- | --- | --- | --- | --- | --- |
| 1. |  |  |  |  |  |  |
| 2. |  |  |  |  |  |  |
| 3. |  |  |  |  |  |  |
| 4. |  |  |  |  |  |  |
| 5. |  |  |  |  |  |  |
| 6. |  |  |  |  |  |  |
| 7. |  |  |  |  |  |  |
| 8. |  |  |  |  |  |  |
| 9. |  |  |  |  |  |  |
| 10. |  |  |  |  |  |  |
